# Vulnerability of the agricultural sector to climate change: The development of a pan-tropical Climate Risk Vulnerability Assessment to inform sub-national decision making

**DOI:** 10.1371/journal.pone.0213641

**Published:** 2019-03-27

**Authors:** Louis Parker, Clement Bourgoin, Armando Martinez-Valle, Peter Läderach

**Affiliations:** 1 CGIAR Research Program on Climate Change, Agriculture and Food Security (CCAFS), International Center for Tropical Agriculture (CIAT), Hanoi, Vietnam; 2 Centre de coopération Internationale en Recherche Agronomique pour le Développement (CIRAD) UPR Forêts et Sociétés, 34398 Montpellier, France; 3 CGIAR Research Program on Climate Change, Agriculture and Food Security (CCAFS), International Center for Tropical Agriculture (CIAT), Managua, Nicaragua; University of Southern Queensland, AUSTRALIA

## Abstract

As climate change continues to exert increasing pressure upon the livelihoods and agricultural sector of many developing and developed nations, a need exists to understand and prioritise at the sub national scale which areas and communities are most vulnerable. The purpose of this study is to develop a robust, rigorous and replicable methodology that is flexible to data limitations and spatially prioritizes the vulnerability of agriculture and rural livelihoods to climate change. We have applied the methodology in Vietnam, Uganda and Nicaragua, three contrasting developing countries that are particularly threatened by climate change. We conceptualize vulnerability to climate change following the widely adopted combination of sensitivity, exposure and adaptive capacity. We used Ecocrop and Maxent ecological models under a high emission climate scenario to assess the sensitivity of the main food security and cash crops to climate change. Using a participatory approach, we identified exposure to natural hazards and the main indicators of adaptive capacity, which were modelled and analysed using geographic information systems. We finally combined the components of vulnerability using equal-weighting to produce a crop specific vulnerability index and a final accumulative score. We have mapped the hotspots of climate change vulnerability and identified the underlying driving indicators. For example, in Vietnam we found the Mekong delta to be one of the vulnerable regions due to a decline in the climatic suitability of rice and maize, combined with high exposure to flooding, sea level rise and drought. However, the region is marked by a relatively high adaptive capacity due to developed infrastructure and comparatively high levels of education. The approach and information derived from the study informs public climate change policies and actions, as vulnerability assessments are the bases of any National Adaptation Plans (NAP), National Determined Contributions (NDC) and for accessing climate finance.

## Introduction

Positive strides have been made at the international level to address the impacts of climate change. The 21st meeting of the Conference of the Parties (COP 21) under the United Nations Framework Convention on Climate Change (UNFCCC) helped mainstream the establishment of nationally determined contributions (NDCs) which bind countries to reach predefined mitigation and offsetting targets [1]. In order to support countries in reaching their respective NDCs, funding for climate adaptation and mitigation projects through mechanisms such as the Green Climate Fund (GCF) have been established. Although an in-balance between the climate finances available and the necessary investments needed to address climate change persists [2], COP 21 contributed to positive engagements, commitments and increased funding for climate change mitigation and adaptation activities [2].

A first step in order to attract bilateral and multilateral support is for countries to prioritize the sub national areas that are most vulnerable to climate change and require technical assistance [3]. Given the extent to which climate change is already affecting many vulnerable communities [4], and the finite resources available to development and governmental agencies [2], it is of paramount importance that interventions are strategically designed and implemented [3]. Vulnerability assessments (VA) have been developed to provide guidance and support for adaptation planning and justification for project implementation in order to create a more objective decision making process [5,6]. Vulnerability assessments can help to identify which communities are the most vulnerable to climate change, where they are located, and what is driving this vulnerability [7].

Agriculture is one of the sectors most vulnerable to changes in the climate [4]. At the global level the Intergovernmental Panel on Climate Change (IPCC) note that yields are generally expected to decline most severely in countries at lower latitudes [8,9]. Recent studies have shown that projected losses in the production of cacao [10] and coffee [11] threaten national economies and also the regional and global supply chains of these respective industries. The projected impacts of climate change are a threat to crop production in regions that currently experience food insecurity [12]. In Africa and south Asia, major grains including wheat, maize and sorghum are projected to suffer mean yield losses of 8% by 2050 with some crops, notably wheat in Africa, expected to experience a yield change of -17% [12]. The impacts of climate change on agriculture are already being felt, in India, climate change induced disturbances to the monsoon recorded between 1966–2002 are estimated to have reduced yields of rice by 4% [13]. Although the impacts of climate change will impact national and global industries [10], it is the marginalised and impoverished rural communities in developing countries whose livelihoods are dependent upon small scale agriculture who are particularly vulnerable [14–17].

Over the past decade a growing number of vulnerability assessments have emerged from the scientific literature that focus on assessing the vulnerability of various sectors, including agriculture to climate change [18,19,20]. A number of studies [21,22,23] have adopted the well-established IPCC [24,9] definition of vulnerability which can be reflected in “the extent to which a natural or social system is susceptible to sustaining damage from climate change impacts, and is a function of exposure, sensitivity and adaptive capacity” [22]. The impact of climate change on agriculture and livelihoods therefore can be conceptualized as the aggregation of these components [19].

The distinction between exposure, sensitivity, and also adaptive capacity (AC) can be opaque. Fritzsche *et al* [19] recommend that exposure variables are strictly climatic phenomena, such as the magnitude, rate of change or variation in rainfall and temperature or meteorological events such as drought or flooding. Smit et al [25] argues that certain variables can be indicative of both exposure and sensitivity and suggests that clear boundaries between the two components do not exist. Whilst Caffrey et al [6] note that sensitivity and adaptive capacity can be combined to indicate “social vulnerability” [20,26]. A recent study by Thiault et al [23] has applied the conceptual framework of vulnerability [27] but through a socio ecological lens in which they quantify the vulnerability of human and marine systems which are intricately connected, using small scale fishing communities in Moorea, French Polynesia as a case study. The ecological vulnerability is combined with the human societal vulnerability in order to spatially prioritise conservation efforts. The study uses primary data collection to characterize the households and develop the socio and ecological vulnerability indices based on the grouping of multiple indicators. Unlike our study the focus is not on a driver (climate change) of change, but rather on assessing how a complex and integrated system can be understood through interconnected vulnerability. Nonetheless, the attempts to include multiple indicators to capture the complexity of vulnerability are shared with our proposed approach. Therefore, what constitutes exposure, sensitivity and adaptive capacity is dependent upon the context of the question and the predefined definitions.

The approach to vulnerability used in this study develops upon the assertions of Turner et al [28] that vulnerability is determined by the degree to which a system will experience stress due to a given pressure or combination of pressures. In our case the pressures are framed within the conceptual framework of vulnerability as a function of sensitivity, exposure and adaptive capacity [7,19,24]. Exposure, is deemed to be the combination of different natural hazards, sensitivity is the projected changes in the climate (precipitation and temperature) and the impacts on climatic suitability for key agricultural crops, and adaptive capacity is the socio-economic standing of the community, and how well they are able to deal with the potential impact (Exposure and Sensitivity).

A number of studies have used spatial analysis and geographic information systems (GIS) to quantify vulnerability to climate change (S1 Table). For example, at the global level vulnerability assessments have been used to identify which countries are more vulnerable to climate change shocks that affect a specific industry, such as fisheries [28]. Several studies have focused on non sector specific vulnerability to climate change but operationalized at a resolution that is sub national thus allowing comparison between and within countries [29,30]. At a similar resolution and coverage, attempts have been made to quantify the vulnerability of agricultural areas to climate change [31,32] and more recently, efforts have focused on the impacts of climate change on a specific crop [23] or combination of crops [7] and the ensuing vulnerability of rural communities.

It has been noted that several studies have projected the future climate and assessed how this influences vulnerability [7,20,33] however, fewer attempts have been made to also project the future societal context of the community (adaptive capacity), as these indicators are often hard to predict and uncertain [6,34]. Likewise, high levels of uncertainty inhibit confidence in the prediction of natural hazards (exposure) which are by nature unstable and chaotic [35]. Thus, our study will not attempt to project changes in adaptive capacity or exposure, but instead assume that current levels of adaptive capacity and exposure will remain constant under the future climate change scenario [6]. We are therefore focusing on how a future high emission climate scenario will affect the suitability of key agricultural crops, and how the exposure to natural hazards and the communities ability to respond (adaptive capacity) under current conditions will manifest itself as vulnerability. The output of vulnerability assessments can be at a resolution equivalent to national [28], sub national [7,31,32] or sub administrative level [23] depending on the context of the study and available data.

In this paper we propose a Climate Risk Vulnerability Assessment (CRVA) methodology that is designed to be flexible whilst providing guidance in regard to conceptualisation of vulnerability, assigned indicators, datasets and methods. We fulfil a gap in the current literature by modelling the impacts of climate change on current and future conditions for multiple crops that dominate the agricultural sector, integrating the impacts of natural hazards into the exposure component and combining the adaptive capacity at a fine scale relevant for policy making. Our objective is to provide a flexible and robust methodology that can be applied to countries across the tropics in order to identify at the sub national scale which rural areas are most vulnerable to the impacts of projected climate change on agriculture.

We tested the CRVA methodology in Vietnam, Uganda and Nicaragua, three developing countries that are particularly threatened by climate change. In the following section, we introduce the case study countries and their main risks to climate change, the selected crops used for the ecological modelling, and the GIS data and its implementation within the spatial model structure to assess vulnerability. We then present the results of each component of vulnerability and the final index. We identify the main hotspots of vulnerability and the underlying driving factors. Finally we discuss the relevant indicators and their integration within the broader discourse on vulnerability assessments that are designed to inform decision making under progressive climate change.

## Materials and methods

### Case study countries to test the CRVA

In order to test the CRVA methodology three countries have been selected: Vietnam, Uganda and Nicaragua. Both Vietnam (7th) and Nicaragua (4) have been placed in the top 10 most affected countries [36] to climate risks over the past two decades (1994–2013). Uganda, a low income [37] landlocked country located in Sub Saharan Africa, has also been identified as a country highly vulnerable to climate change [6,38,39]. All three countries have a substantial agricultural sector; as of 2016 agriculture comprised 16.3% of GDP and employed 48% of the labour force in Vietnam [40], in Nicaragua 15% of GDP is attributed to agriculture and employs 31% of the labour force [41], and in Uganda 25% of GDP is derived from agriculture, and 72% of the labour force are employed in agriculture [42].

#### Vietnam

In Vietnam progressive climate change will lead to increased temperatures and altering precipitation patterns, resulting in impacts such as rising sea levels, a higher probability of floods and droughts and more intense tropical cyclones [43,44]. In 2016, Vietnam was in the top ten countries in regard to number of people affected by natural disasters [45]. This can impact upon agricultural production and rural livelihoods, for instance flooding has been found to affect arable land in Quang Nam province, Vietnam and impact up to 40% of wet rice production [46].

#### Uganda

Climate change will likely increase food insecurity in Uganda and the impacts are likely to be particularly detrimental for the production of coffee, with estimates that the entire sector could be lost within the next 30–70 year [38].

Uganda is one of the ASAP-IFAD countries for investment [47]. Under its Vision 2040 Uganda seeks to: Integrate climate change issues (mitigation and adaptation) in all government plans and programs as a key development factor [48].

#### Nicaragua

Nicaragua is the poorest country in Central America [41] and has been identified as highly vulnerable to climate change [7] especially for coffee [49], maize and bean [50] which are important cash and food security crops [51]. The ongoing ASAP IFAD [52] project is addressing these challenges and the here presented project will support with CSA prioritization and economic valuation tools.

### Selection of the main food security and cash crops

In order to prioritize which crops to include in the CRVA (Table 1), we used an existing methodology [53] which identifies and prioritizes which crops are most important to food security for a given country, based on the following set of indicators: harvested area; net production value (NPV), gross production value (GPV), contribution to national and agricultural gross domestic product (GDP) and calories intake (kcal/ capita/ day). These indicators help establish the relevance of the crop production system for the country’s economy and food security. As a first option to collect this data we used the national statistics census and in the case that is does not exist or is not available we used FAOSTAT [54].

**Table 1:** Selected crops for Nicaragua, Uganda and Vietnam based on chosen indicators to capture cash crops and food security.

Country	Crop	Net Production Value (constant 2004–2006 USD millions)	Gross Production Value (constant 2004–2006 USD millions)	Production system contribution to agricultural GDP (%)	Production system contribution to national GDP (%)	Food supply (Kcal/capita/day)	Harvested Area (Ha)
Nicaragua	Coffee Arabica	96	96	3.68	1.09	0.66	116,129
	Bean	126	132	4.93	1.48	178	202,565
	Rice	111	111	4.20	1.25	405	92,832
	Maize	63	71	2.71	0.80	629	271,514
	Cocoa	2	2	0.067	0.02	5	6,277
Uganda	Plantain	1,449	2	33.32	9.24	327	1,689,270
	Cassava	530	530	9.12	2.53	275	590,830
	Maize	321	362	6.18	1.71	332	1,046,400
	Bean	239	270	4.66	1.30	97	840,292
	Sweet potato	202	202	3.49	0.97	179	532,958
Vietnam	Rice	10,000	12	NA	NA	1388	7,647,602
	Coffee Robusta	1,389	1,389	NA	NA	-	544,033
	Maize	NA	703	NA	NA	90	1,125,078
	Cassava	1,007	1,007	NA	NA	22	531,778
	Cashew	744	744	NA	NA	19	326,768

The table reports numbers for Net Production Value and Gross Production Value rounded to the nearest million.

The indicators were calculated based on a five year average time period (most recent years with available data). A 5 year time period captures long term trends [55] and what the FAO defines as “chronic food insecurity”, in which persistent and long term stresses regarding the definition of food security are present, as opposed to “Transitory Food Insecurity” which is short term, annual variation and often characterized by sudden economic, cultural, political or natural shocks that temporarily and suddenly reduce the availability or access to nutritional food sources [56].

In the case of Uganda and Vietnam all indicators were collected from FAOSTAT [54]. For Nicaragua we use data from FAOSTAT [54] except for contribution to agricultural and national GDP which comes from the Central Bank of Nicaragua [51].

### Spatial data collection

The GIS data which was employed as part of the CRVA in Nicaragua, Uganda and Vietnam is organized by scale from global to sub-national and displays for each component of vulnerability; selected variables, a brief description of how the data can be interpreted, the source and the spatial resolution (Table 2). The strength of this approach was to collect mainly free and open source data at the finest spatial resolution possible with the restriction to cover the full country extent.

**Table 2:** Descriptive information on the indicators and data used for the three case studies.

□Nicaragua ◊Uganda ○Vietnam					
*Scale*	*Components of vulnerability*	*Indicator*	*Description*	*Source of data*	*Resolution*
*Global*	**Sensitivity**	Current climatic data □◊○	Current temperature and precipitation dating from 1950 to 2000. Identify areas projected to experience greatest change in temperature	WorldClim [57]	2.5 arc minutes (~5km)
		GCMs projected data □◊○	Long term projection of climate (2040–2069 representing 2050 decadal time period). Identify areas projected to experience greatest change in precipitation	GCM [58]	2.5 arc minutes (~5km)
	**Natural Hazards**	DEM	Digital Elevation Model◊	NASA SRTM [59]	1 arc second (30m)
			Corrected DEM (filled no-data voids by interpolation) ○	Jarvis et al. [60]	90 meters
		Land cover	Land cover classification (2010) ◊	Chen et al [61]	30 meters
			MODIS-based Global Land Cover Climatology describes land cover type, and is based on 10 years (2001–2010) of Collection 5.1 MCD12Q1○	Broxton et al [62]	500 meters
		Flooding□◊○	Flood events	UNEP [63]	0.0083 degrees (~1km)
		Drought□◊	Aridity is the ratio of the mean annual precipitation and the mean annual potential evapo-transpiration	CGIAR-CSI [64]	30 arc seconds (~1km)
		Fire◊	Fire hotspots	MODIS fire product [65]	1km
		Sea level rise○	> _ mm of sea level rise	Li et al [66]	1km
		Tropical cyclones□○	>_ hurricane events per _ years of an intensity	UNEP [67]	0.0173 degrees (~2km)
		Soil data○	Harmonized World Soil Database	FAO [68]	30 arc seconds (~1km)
		Road system□◊○	Road shapefile	The Digital Chart of the World [69]	1:1,000,000 scale vector
*National*	**Natural Hazards**	Land degradation◊	Weighted combination of vegetation cover and quality, rainfall erosive, slope factor, soil erodibility and population density.	Monitoring for Environment and Security in Africa (MESA) [70]	100 meters
		Soil erosion□	This map was generated based on a qualitative evaluation during a survey of soils.	Agricultural Ministry of Nicaragua [71]	1:50000 scale vector
		Drought○	Average days of drought in a year period based on a 5 years average period (2007–2012)	ISS [72]	0.033 degrees (~3687m)
*Sub national*	**Sensitivity**	Harvested area data○	Harvested area data at the finest scale (province and/or district) for each modelled crop.	Vietnam General statistics Office [73]	Province vector shapefiles
	**Adaptive Capacity**	Education	Primary net intake rate◊	Uganda Bureau of Statistics [74]	District shapefile
			Percentage of graduates compared with total upper secondary candidates○	Vietnam General statistics Office [75]	Province shapefile
		Poverty	GINI index◊○	Uganda GINI index, [76] National Economics University [77]	Subcounties (Uganda) District (Vietnam)
		Organizational Capacity○	The number of agricultural cooperatives [78] over the number of total farms [79]	Vietnam General statistics Office	Province shapefile
		Health care	Average of underweight, stunting and wasting of total population○	National Institute of Nutrition [80]	Province shapefile
			Average of the ratio of the number of health facilities by population, average immunization rate for 4 major antigens, latrine coverage in households, per capita outpatient department utilization in government and private not for profit (PNFP) health and deliveries in government and PNFP health facilities◊	Uganda Bureau of Statistics [74]	District shapefile
		Accessibility/infrastructure◊○	Travel time in hours to urban areas. Input maps divided into target locations (populated places) and Friction surface (Road Network, Railway Network, Navigable rivers, Major waterbodies, Shipping lanes, National Borders, Landcover, Urban areas, Elevation, Slope	Nelson [81]	30 arcs–seconds

### Vulnerability assessment

#### CRVA general approach

**Fig 1:**
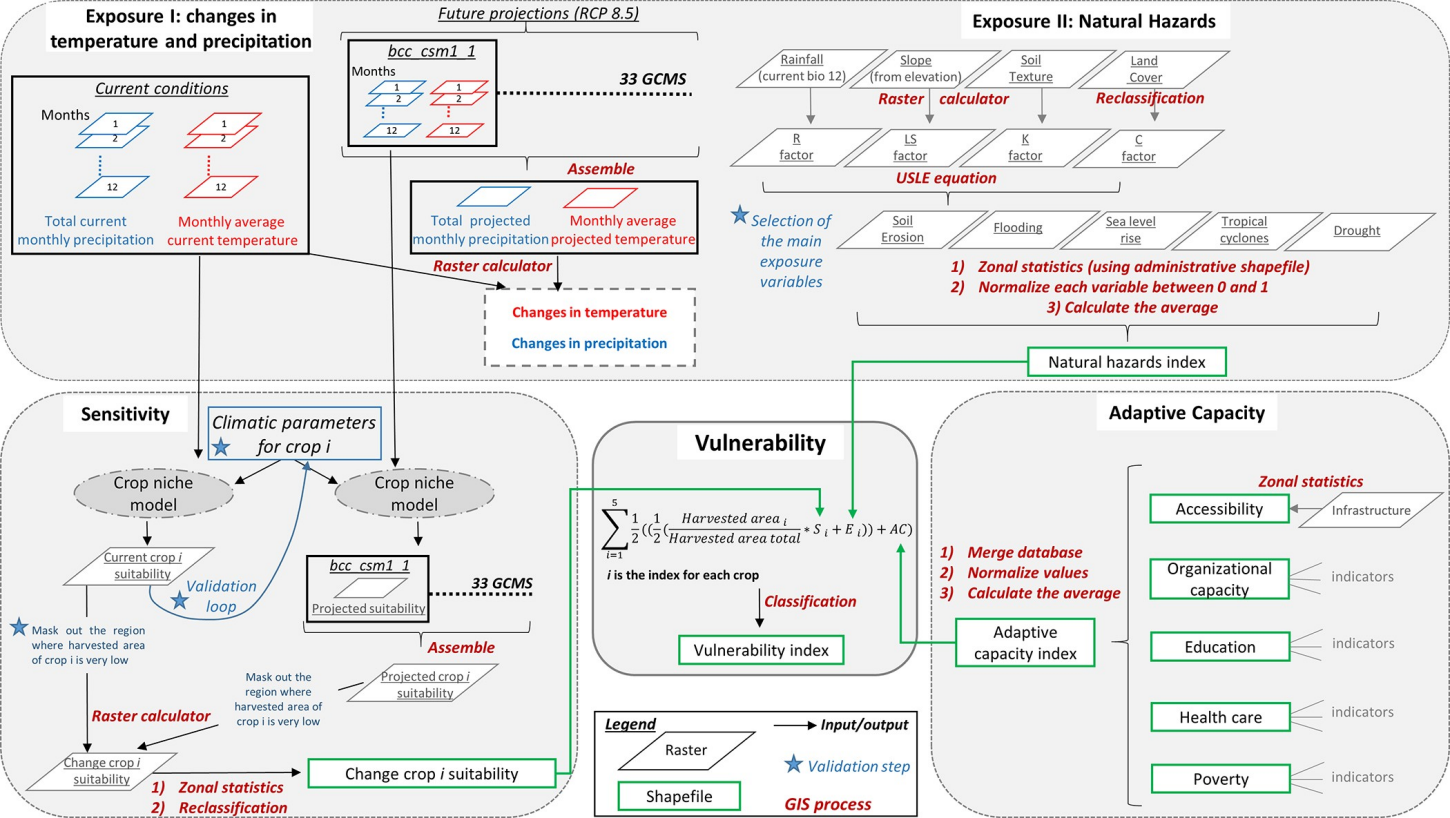
A Framework to assess the vulnerability of agriculture and rural livelihoods to projected climate change. **Source: Adapted from Marshal et al (2010) [27].** The framework is divided into four main grey boxes in which the outputs are combined into the final vulnerability index. Black arrows indicate the direction from input to output for the GIS process labelled in red. Rectangular green boxes indicate the output of the GIS process, which are formatted into shapefile datasets. Raster spatial data are displayed by grey parallelograms except for the climatic rasters where blue and red colours refer respectively to current and future conditions. Finally, blue stars refer to the requirement for expert validation or input from the scientific literature.

In this section we display (Fig 1) how the GIS multiscale data has been aggregated within the three components of vulnerability to obtain a single index of vulnerability based on the combined cropping systems. Each step of the process that requires geo spatial analyses has been highlighted in the framework below.

#### Exposure assessment

We divide exposure into two components: exposure 1 refers to the changes in temperature and precipitation between current conditions and the future projection (decade 2050). To estimate the current conditions we use Worldclim, a set of global climatic layers that utilise weather stations from sources such as the Global Historical Climatology Network (GHCN) and FAO, with records dating from 1950 to 2000 [57]. Using the thin plate spine algorithm [82] the climate data has been interpolated at a spatial resolution of 2.5 arc-minutes (~ 5 km^2^ at the equator) for monthly total rainfall, and maximum, mean and minimum temperature. For the future climate data we use the Representative Concentration Pathway 8.5 (RCP), characterised by increasing green-house gas emissions, high rates of population growth, modest GDP growth and low rates of technological development and uptake [83]. This is the most severe (~1370 ppm CO_2_ eq by 2100) of the future scenarios which were documented in The Fifth Assessment Report [4] of the IPCC. The future period selected for this study is the decade 2050 (representing 204–2069), corresponding to a medium term horizon [84]. In order to simulate the future climate and atmosphere we used the mean ensemble of 32 General Circulation Models (GCMs) under RCP 8.5. The spatial resolution of GCMs is too coarse to analyse the direct impacts on farmers’ production. We therefore used downscaled data based on the sum of interpolated anomalies for each GCM against the current climate baseline data [85], creating a smoothed climate surface at 2.5 arc minutes for future climate conditions. The changes in precipitation and temperature were estimated by subtracting current to future climate, using the downscaled datasets in order to describe changes in temperature and precipitation.

Second, exposure 2 includes natural hazards (sea level rise, flooding, drought and tropical cyclones) and hazardous processes (soil erosion and land degradation) which are deemed to present significant risk to the agricultural sector [86] and have been used in previous studies to develop disaster risk and vulnerability indexes [87,19]. We do not model the aforementioned hazards and instead rely on previous studies that developed spatially aggregated datasets (Table 2). A number of those included (Table 2) are acquired from the Global Data Risk Platform [88] which provides spatially referenced hazard data at a high resolution and extensive coverage. In regards to flooding and drought, the climate risks are derived from historical records and statistical modelling that spatially infers extreme events that go beyond the natural variability in the system [89]. Due to the different geographical and meteorological contexts of the respective case study countries, the selection of which natural hazard variables to include requires literature review and validation from local experts. The approach to Exposure 2 varied between the three case studies. In Vietnam, we undertook a workshop in Hanoi, attended by agronomists from the Consultative Group for International Agricultural Research (CGIAR), representatives from our government partners, notably the Institute of Agriculture and Environment (IAE) and the National Institute of Agricultural Planning and Projection (NIAPP), the Institute of Geography from the University of Science and Technology also attended, as did a number of non-governmental organizations including the Cooperative for Assistance and Relief Everywhere (CARE) and the Food and Agricultural Organization (FAO). The attendees brought a variety of focuses and expertise that covered the length and breadth of Vietnam and this was necessary to ensure accurate validation of our input data. During this workshop we undertook an exercise with the participants to confirm which of the natural hazards (exposure) affects each of the respective crops (S1 File). The natural hazard data is spatially explicit and covers the full extent of Vietnam (Table 2). We used this information to create exposure specific indexes for each of the crops. The purpose was to develop a more refined Potential Impact indices by focusing on the relevant natural hazards that affect the respective cropping system. In the case of Uganda and Nicaragua, meetings were undertaken with experts and government partners to identify natural hazards and also identify suitable datasets, but due to limited access to agronomists on the respective crops, the crop specific exposure index was not calculated. Therefore, we assumed that the respective natural hazards (Table 2) affected all cropping systems equally.

In the case of Vietnam we estimated potential soil erosion based on the Universal Soil Loss Equation (USLE). We calculated the potential erosion using available data from different sources (S2 Table) and combined it by using raster calculator in ArcMap 10.3. In the case of Nicaragua we used the pre-existing erosion map [71] whilst for Uganda we used an existing land degradation map [70].

In order to calculate the exposure index for Uganda and Nicaragua we extracted the values of each of the natural hazards with the administrative boundary shapefile, rescaled all the values between 0 and 1 and calculated the average of all natural hazard variables at the administrative scale for the full extent of the country (Fig 1). As mentioned, in the case of Vietnam we created a crop specific exposure index, this involved undertaking the same process of extracting and rescaling the natural hazards from 0 to 1, but the average is confined to those natural hazards that affect the respective crop. Thereby, in Vietnam we created one exposure index for each crop whilst in the case of Uganda and Nicaragua we have one exposure index applied to all respective crops.

#### Sensitivity assessment

For this study sensitivity is understood as the change in the climatic suitability of an area to grow a crop. We estimated this change by subtracting the current climatic suitability from the future suitability. We used the MaxEnt Model [90] for the modelling of coffee and rice, as the model [90] performs well for crops that are often irrigated (rice) or grow at particular elevational ranges (coffee Arabica and Robusta). In Vietnam we are focused on coffee Robusta, whilst in Nicaragua we are concerned with coffee Arabica (Table 1). For modelling of maize in Vietnam we used the Ecocrop model, a crop niche prediction model with the same name as the Food and Agriculture Organization (FAO) Ecocrop database [91]. The basic model uses environmental ranges [92] as inputs to determine climatic suitability. S1 File displays which model was used to simulate current and future crop suitability across our three case study countries.

In order to calibrate the model and ensure valid results we rely on a combination of academic literature, government statistics and expert feedback. We first produce climate suitability maps for the respective crop under current conditions. We identify an expert who understands and works with the respective crop and we request feedback on the accuracy of the climate suitability map for the respective crop and the input parameters used. In certain cases the expert will identify areas where the model is not performing well, we subsequently re-run the model and send the updated results, we repeat this process until the current climate crop suitability maps capture the current distribution of the respective crop. Feedback on the crop parameters and maps are crucial in order to improve the validity of the sensitivity mapping. Once the current suitability maps have been approved by experts and compared to national production statistics and the wider literature, the future data (2050), using 33 GCMs with RCP 8.5 climatic data can be used as inputs for the niche crop model to generate 33 projected suitability outputs. From this, we calculate the average and the standard deviation in order to analyse the variability of the GCMs. Another validation step is to mask out the regions with low production data or when current suitability values are lower than 20%. We finally calculate the change between current and projected suitability, extract the values for each administrative unit and classify them into a sensitivity index (Table 3):

**Table 3:** Index used to capture change in the climate suitability for respective crops under climate change scenario for 2050.

Classification	Changes (%)	Sensitivity Index
Negative	-50 - -100	1
	-25 - -49	0.5
	-5 - -24	0.25
No change / no crop presence	- 5 - +5	0
Positive	5–24	-0.25
	26–49	-0.5
	50–100	-1

In the table, changes refers to the % change in crop climate suitability from current to future (2050) conditions for the respective administrative unit. Sensitivity index is the score attributed to the change and is classified as negative, no change, or positive.

#### Adaptive capacity assessment

For the adaptive capacity component we compiled shapefile datasets (Table 2) for each of our respective indicators (education, poverty, organizational capacity, health care, accessibility/ infrastructure) and normalized all values from 0 to 1. In the case of poverty, we inverted the gini coefficient index so that values of 1 signified total equality, whilst a value of 0 indicated maximal inequality. This is because we assume that high levels of inequality equate to low adaptive capacity. In regard to the health indicator (Table 2) we created an average of the input datasets and then normalized from 0–1, we subsequently inverted the data so that values near to 1 indicated positive health care provision, whilst a value near 0 suggested low health care provision. For example, in the case of Vietnam the districts with high values for health care possessed less incidence of underweight, stunting and wasting (S1 Fig). Once all the shapefile indicators are normalized from 0–1, we calculate the average, thereby producing a final adaptive capacity index where 0 equates to no adaptive capacity and a value of 1 indicates absolute adaptive capacity.

The adaptive capacity index’s resolution corresponds to the finest resolution of all the indicators used and drives the resolution of the overall vulnerability output. For each variable of adaptive capacity, there are many indicators that can be used depending on data limitation or the adopted definitions in the country of interest. In the case of Vietnam (S1 Fig), adaptive capacity is divided into 5 variables (Table 2); whilst in Uganda a total of 4 variables were included, as a lack of data on organizational capacity prevented its inclusion. The datasets used to capture adaptive capacity cover the full extent of the respective country and are available at a resolution that enables sub national analysis (Table 2). In the case of Nicaragua adaptive capacity has been calculated based on data generated by Bouroncle et al. [7]. In the aforementioned study adaptive capacity was mapped as a function of three conditions: satisfaction of basic needs, resources for innovation and resources for transforming innovation into actions. A series of indicators were compiled (S3 Table). The indicators were normalized with values linearly to a 0–1 interval based on their minimum and maximum values in each municipality in order to avoid biases due to wide variations of socioeconomic development [7].

#### Final vulnerability index assessment

We finally use the combination of the normalized natural hazards, adaptive capacity and sensitivity (crop specific) to calculate the overall vulnerability at the administrative boundary scale described before. When data on harvested area for each crop is available (Table 2), it is possible to weight the sensitivity component by the ratio of harvest area of that particular crop to the total harvested area of the cropping system. We used this weighted equation for Vietnam in order to give more importance to the final overall vulnerability to prioritise where the crop is present and grown in greater quantities (harvested area). Data for harvested area was not available for Uganda or Nicaragua at the sub county level. We present the equation used to calculate the overall vulnerability in Vietnam. The analysis was conducted in ArcGIS 10.3 software (Environmental Systems Research Institute, Redlands, California). The overall vulnerability is determined by equally weighted contribution of potential impact (*S*_*i*_+*E*_*i*_) and Adaptive Capacity (*AC*) [27].

$$Overall\;vulnerability=\sum_{i=1}^{5}\frac{1}{2}((\frac{1}{2}(\frac{Harvested\;area_{i}}{Harvested\;area\;total}*S{}_{i}+E{}_{i}))+AC)$$

Where: *i* = Each of the crops, *Harvested area*_*i*_ = Harvested area per crop, *Harvested area total* = Total harvested area for all 5 crops, *S*_*i*_ = Sensitivity of the crop _i_, *E*_*i*_ = Exposure of the crop _I_, *AC* = Adaptive Capacity.

#### Validation and dissemination

In the case of Vietnam and Nicaragua expert validation was obtained at various stages of the vulnerability methodology (Fig 1). In Vietnam a planning workshop with government partners, agronomists and stakeholders provided feedback on the indicators (datasets) used to determine the components of vulnerability. In the case of Nicaragua experts were consulted in order to obtain the indicators of adaptive capacity. In the case of Uganda, time constraints and data limitations meant that the same level of scrutiny of the data was not undertaken. Therefore the results for Uganda should be shared with experts in order to gauge the reliability of the input data. Finally, the combination of the indicators within the three components of vulnerability was undertaken without weighting due to a lack of production data, expertise on the importance of each indicator and limited feedback from crop experts.

## Results and discussion

**Fig 2:**
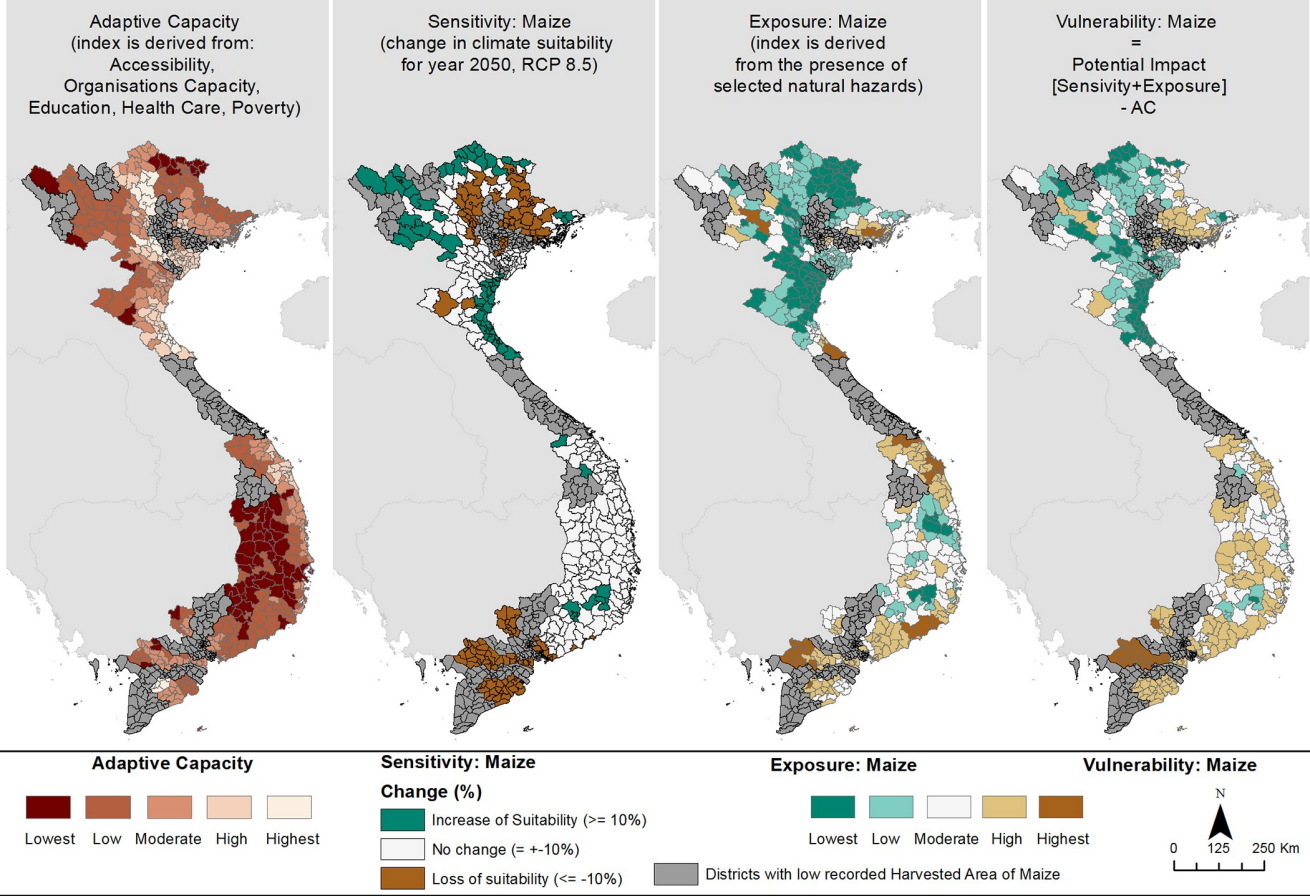
Vulnerability of maize to climate change (2050) under a high emission scenario (RCP 8.5).

In the following section we will present in detail the results of the CRVA for Vietnam using maize as an example to display the adaptive capacity, sensitivity, exposure and overall vulnerability of the cropping system to climate change (Fig 2). Although we will focus on maize, the overall vulnerability index for rice and coffee Robusta in Vietnam are displayed in S2 Fig. We will then present the final vulnerability index for each country and highlight for Vietnam and Nicaragua, where expert validation has been provided, three areas of comparatively high vulnerability and the indicators driving the elevated scores.

Adaptive capacity for the maize growing areas is lowest in the central highland areas, and also in Muong Te district in Lai Chau province (north western Vietnam), additionally, Bao Lac, Ha Quang and Trung Khanh districts, in Cao Bang province also possessed low AC. In regard to sensitivity a number of districts in the Mekong delta and also northern regions of Vietnam experienced a decline in suitability. Exposure for maize was highest in the Mekong delta, due to the presence of flooding and drought, the central coastal zones due to the presence of tropical cyclones, flooding and also drought, and finally in Muong la and Quynh Nhai district (Son La province), due to soil erosion risk and flooding.

Maize is important in Vietnam both as a cash crop, as it is used as a feed for the poultry and livestock industry, and for food security, especially in mountainous areas where it has in the past replaced rice in times of shortages [93,94]. The overall vulnerability map for maize suggests that the districts in the Mekong river delta are vulnerable to climate change, due to a loss in the climatic suitability of maize, the presence of multiple natural hazards (flooding, drought and some expected sea level rise) and also the relatively low AC in certain districts. Additional districts of high vulnerability were found in Dong Bac province (north east Vietnam) and Dong Nam province in central south eastern Vietnam. The overall vulnerability map for maize can be used to stimulate conversation amongst interested stakeholders and policy makers in regard to investing in climate change adaptation strategies.

**Fig 3:**
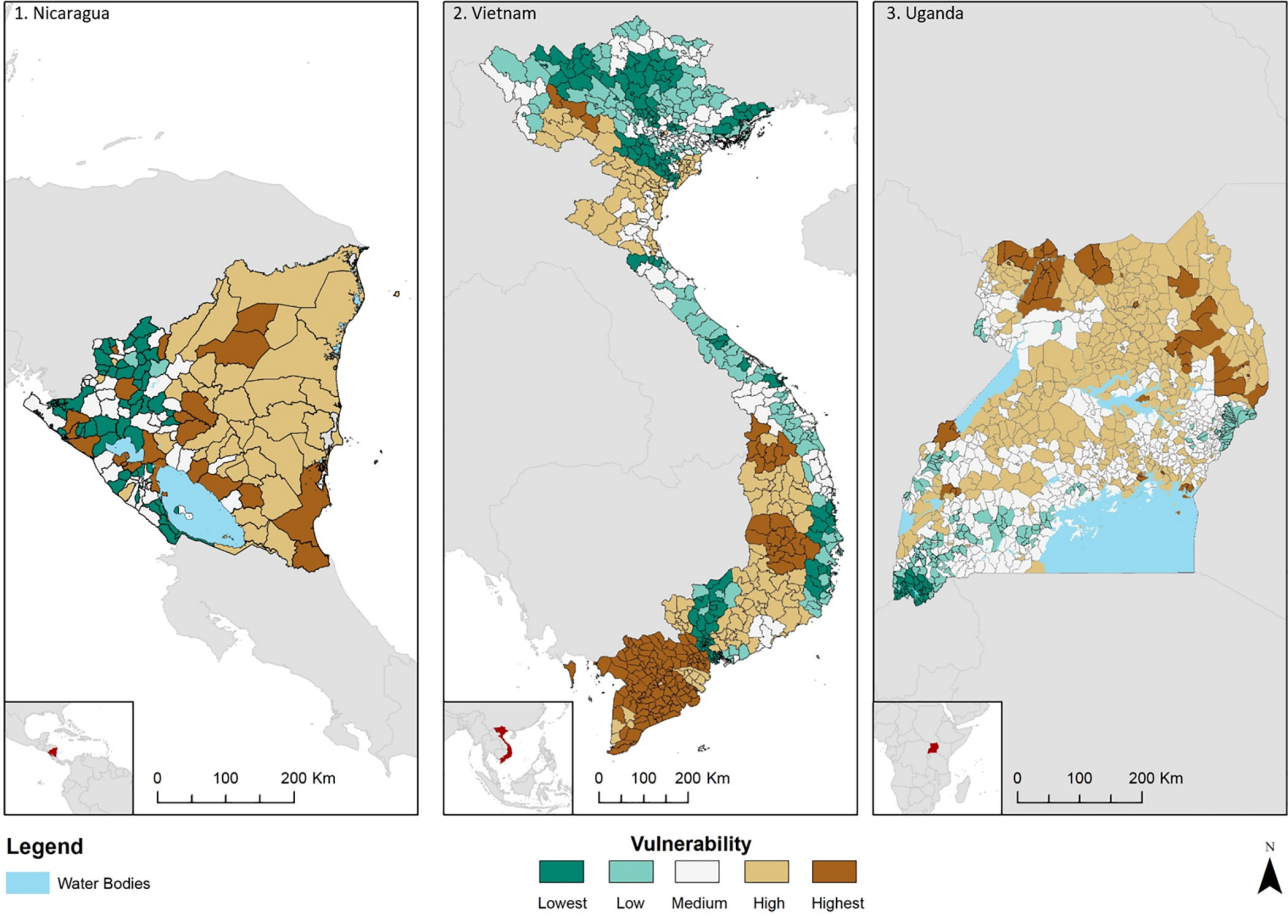
Vulnerability to climate change (2050) under a high emission scenario (RCP 8.5) for Nicaragua, Uganda and Vietnam, calculated as a function of exposure to natural hazards, sensitivity of selected crops to climate change and adaptive capacity of the population.

Finally, we have calculated an overall vulnerability index based on the aggregate of the respective analysed cropping systems in each country. Fig 3 highlights the relative and country specific vulnerability of the agriculture sector for each of the countries to climate change.

The causes of vulnerability can be attributed to different drivers and are specific to each country (S4 Table). Vulnerability in Nicaragua was found to be high in a number of municipalities, notably Wiwili de Nueva Segovia (region 1), Siuna and Bonanza (region 2), and Rancho Grande, Matiguas, Muy Muy and Boaco (region 3). In the case of Vietnam we also identified three regions of high vulnerability, notably: the north west (region 1), the central highlands (region 2) and finally the Mekong Delta (region 3). These regions for both case studies are displayed on the final vulnerability index map (Fig 3). The drivers behind the vulnerability vary for the respective regions. For instance, the Mekong Delta, Vietnam, was identified as vulnerable to climate change due to the loss of climatic suitability for rice and maize, combined with high exposure to flooding, sea level rise and drought (S4 Table).

Some areas in Vietnam are less vulnerable than may have been expected. For example, the overall vulnerability of districts in Ha Giang province, north Vietnam, are generally low or medium (Fig 3). This can be attributed to relatively high scores in adaptive capacity, contributed to by strong scores in organisational capacity and also medium range scores in education (See Adaptive Capacity map in S1 Fig). Additionally, the impacts of climate change on the analysed crops are projected to be less severe in this region, for instance, in the case of Qung Ba district (Ha Giang province) only maize is expected to become less climatically suitable (-0.25%). Additionally, the score for exposure is generally comparatively low in Ha Giang. Sea level rise, tropical cyclones and to a lesser extent drought and flooding were not threats according to the data we used in the study. Future studies could include additional natural hazards such as land-slides which may affect the results and increase the relative vulnerability of mountainous areas, including Ha Giang.

### Indicators for the CRVA

The following section will discuss the indicators used to capture vulnerability as part of this study. It is important to understand that vulnerability is not a measurable phenomenon, it is instead a dynamic state which is the result of multiple interacting variables [19]. As vulnerability cannot be measured directly [95], a number of indicators which are presumed to affect vulnerability are aggregated to provide an indication, or an index of vulnerability. This is a relative scale which shows the spatial distribution of vulnerability within the respective unit of analysis, which in our case is the respective countries. The vulnerability cannot be compared between the countries as they use different indicators, instead, one can deduce a sense of which areas at the sub national scale are more or less vulnerable within the country of analysis.

The CRVA provides a list of indicators for exposure, sensitivity and adaptive capacity (see Table 2 in methods) that can be used to determine the vulnerability of a location, at the national, regional, or local scale. As noted by Hinkel [96], there is a huge amount of inherent complexity when assessing vulnerability, with the economic, environmental and socio-economic fabric of the particular country determining which indicators are relevant. Schroter et al [90] has highlighted the need to select indicators which would be understood by the stakeholders, as an emphasis should be placed on science that is robust but also understandable for the policy maker or the end target audience. Hinkel [97] note that vulnerability assessments are often devised to inform policy rather than advance knowledge in an intrinsic sense and Erikson and Kelly [98] noted that data limitations regarding quality and also availability direct the selection of indicators. In spite of the clear data limitations in some regions, Fussel and Klein [99] note that there is a move within vulnerability assessments to include more variables in an attempt to capture the complex realities which determine vulnerability. Schroter et al [79] however, note that care must be taken when choosing which indicators are important, for instance, the same indicator may not be relevant for countries with varying levels of development. Setting the indicators therefore requires an understanding of the country wide drivers of vulnerability, which can be achieved through consultation of the wider literature, discussion with national experts and meetings with stakeholders [17].

The selected indicators (Table 2, see methods) we chose are commonly used measures of agricultural vulnerability [19]. The indicators capture both the regions of high biophysical and climate risks (Exposure), resilience of the crop production systems (Sensitivity) and societal capacity to respond (Adaptive Capacity). The indicators for exposure are natural hazards (sea level rise, flooding, drought, tropical cyclone) and hazardous processes (soil erosion). These indicators have been included in previous multi hazard vulnerability assessments [100].

In respect to adaptive capacity, the indicators listed in Table 2 have been recommended in previous vulnerability methodologies [19]. It is important to note that in the case of Vietnam and Uganda we used indicators from Table 2 for adaptive capacity, whilst in the case of Nicaragua (S3 Table), the adaptive capacity index was based on the livelihoods approach from Bouroncle et al [7]. In order to capture the respective adaptive capacity in Vietnam we focus on the education, poverty, health care, organizational capacity and accessibility/ infrastructure of the particular administrative unit, whilst in Uganda we use the same indicators with the exception of organizational capacity. It is important to note that we have not undertaken household surveys or primary data collection and instead rely on readily available and pre existing datasets that are aggregated to the administrative unit and cover the full extent of the respective countries (Table 2). Due to data limitations a degree of flexibility is required in order to represent these indicators. For example, in regard to education we use the ‘Primary net intake rate’ in Uganda whilst in Vietnam we use the ‘Percentage of graduates compared with total upper secondary candidates’, both datasets were obtained from the respective government statistics office, cover the full extent of the country and are at a high resolution (Table 2). In respect to health care, in Vietnam we created an average of the underweight, stunting and wasting datasets [73] which were available at the province level for the entirety of the country. These input datasets were developed as part of a 2010 National Nutrition Survey [73]. In respect to Uganda, data on the health of the population was derived from a government dataset [69] that captures both access to health care, sanitation and immunization at the district level across the full extent of Uganda (Table 2). In regards to organizational capacity we were not able to obtain reliable data for Uganda. In Vietnam, we used government data at the province level for the number of agricultural unions over the number of farms for the year 2005 (Table 2). Both in Vietnam and Uganda, poverty was represented by the Gini income inequality index, whereby the administrative units with greater levels of inequality were deemed to have lower adaptive capacity. Finally, the accessibility/ infrastructure indicator was derived from a global dataset [74] that estimates the travel time in hours from urban areas (>50,000 inhabitants) based on a number of friction layers (road networks, railway networks, navigable rivers, major water bodies, shipping lanes, national borders, land cover, urban areas, elevation, and slope) at a resolution of 30 arc seconds (Table 2). We calculated an average travel time in hours for the respective districts in Vietnam and Uganda using our administrative shapefile and the raster dataset [74]. The districts with greater travel times were deemed to have lower adaptive capacity.

To summarise, data for the exposure and sensitivity indicators are freely available to download at national scales (Table 2, see methods). Adaptive capacity data for Uganda and Vietnam was attained from government sources (Table 3), whilst in Nicaragua, as noted, data from a previous study was utilised [7]. In regard to selecting datasets to represent the indicators we sought open source data for the most recent year available, at the finest resolution, and covering the full extent of the country. The datasets used are obtained from a variety of sources and are specific to the case study countries (Table 2).

### The novel aspect of the CRVA

The CRVA builds upon previous studies [29,32] which integrate socio-economic, geographical and biophysical data into the vulnerability concept. Nevertheless, the integration of multiple natural hazards such as soil erosion, sea level rise and meteorological stresses such as flooding, tropical cyclones and drought into climate change vulnerability assessments focusing on agriculture remains novel [18]. The CRVA attempts to address this gap in vulnerability assessments through integrating a broader list of natural hazard stresses within the framework. In our study we develop upon the recent work of Bouroncle et al [7], which provided a detailed assessment of vulnerability of the agriculture sector in Central America to climate change, integrating multiple crops and the assessment of adaptive capacity through the livelihoods approach. Hence, the CRVA builds upon this recent study, using a similar methodology but with the inclusion of the natural hazards component within the vulnerability framework. A study by Yusuf and Fransisco [29] undertook a vulnerability assessment for South East Asia which included hazard indicators, but at a coarse resolution and without a focus on agriculture. Nonetheless, studies such as Cutter et al [101] have stressed the need to integrate the social vulnerability with natural hazard risk. Therefore the CRVA answers these calls and offers a more integrative approach through combining natural hazards within agricultural vulnerability to climate change.

The CRVA can be implemented in locations lacking comprehensive national data, as was recently displayed in the Union of the Comoros, with global and regional datasets integrated to provide an insight into the vulnerability of key agricultural commodity value chains to climate change [102]. The CRVA framework integrates global, regional and when available national datasets from a range of sources (Table 2: see methods). The utilised datasets are open source, published, and at a resolution that enables sub national level analysis. Furthermore, the vulnerability methodology enables the inclusion of the most important crops for food security. This goes beyond previous studies [23] which focused on single crop systems. The CRVA proposed therefore provides a more complete understanding of overall agricultural vulnerability to climate change.

Improvements in spatial data provide constantly evolving opportunities to assess vulnerability at the sub national scale. Nevertheless, despite the current “Big Data” revolution in agriculture [103], a lack of datasets with sufficient geographical coverage and historical records to undertake dynamic vulnerability assessments looking at more complex interaction between variables persists [18]. Calls to understand and capture the interaction between variables which determine vulnerability is inhibited not only by a lack of data but also an understanding of the interaction between variables [18]. The CRVA proposes a simplified approach, in that it does not consider dynamic interactions between variables.

When undertaking a spatial vulnerability assessment it is necessary to identify a research area and the unit of analysis. Fekete et al [104] definition of sub national, referring to the administrative unit at which government agencies allocate resources has been used. The “research area” for our study is the respective country. Fekete et al [104] note that policy makers need results at a unit which they understand. The aim is to provide outputs which are useful for policy makers. Therefore a review of government literature and available datasets, in conjunction with input from policy makers and local experts is recommended, in order to understand the administrative structure of the respective country and identify a unit of analysis that respects both data limitations and policy decision and planning structures.

Adopting recognised administrative units is one mechanism to help ensure that the output from vulnerability assessments can be integrated into strategic government plans. Fritzsche [19] notes that information that is generated at a policy relevant administrative level can empower decision makers and non-governmental organisations to more effectively direct human and monetary investment to vulnerable communities most likely to be affected by climate change.

### Uncertainty and decision making

In our study we have adopted the assumption [7,33] that vulnerability is equal to: 50% potential impact (sensitivity and exposure) and 50% adaptive capacity. It is possible that greater weight could be attributed to adaptive capacity, if for instance, human capital rather than the presence of natural hazards (exposure) or loss of climatic suitability for cropping systems (sensitivity) drives vulnerability. Future studies could focus on the impacts on vulnerability of increasing the weight of AC for instance. Furthermore we aggregated the indicators within the three components of vulnerability using a deductive approach [105] with multiple indicators that are thought to influence vulnerability selected and weighted equally. It is possible however to prioritise indicators [106] based on perceived importance which can be achieved in a number of ways, including stakeholder meetings or expert consultations [19].

Datasets produced at a global extent may not effectively capture the sub national context. A recent investigation by Trigg et al [107] tested 6 global models used to derive flood hazard and found that agreement between the models for the African continent was roughly 30–40%, thus highlighting the discrepancies between models when extracted to a reduced coverage. The global dataset for flood frequency [63] used in our study possesses a fine resolution (1km) and was validated by experts through a workshop in the case study countries, Vietnam and Nicaragua. However, there is a chance that at very fine resolutions (<1km) and in areas where flash floods, complex topography, peri-urban landscapes and varying technological and management capacity exist; discrepancies between the model and reality are likely [107]. Additionally, the datasets are historic snapshots and may not capture the current trend, for instance, the flooding data [63] is based on records from 1999–2007. However, this was the most up to date and robust data available at the time of the study. Despite inevitable constraints, the flooding and additional hazard datasets (Table 2, see methods) used in our study have been tested and published [108] and the utilised data has been scrutinised throughout the methodology process (Fig 1, see methods).

The climate suitability modelling results for the current conditions have been verified by experts. They largely capture the known locations where the crop is grown. However, there is uncertainty attached to the future projections of climate suitability which are based on a single RCP (8.5) [92] and are dependent upon the ensemble of 33 GCMs. Using a mean of the GCMs has been adopted to reduce the uncertainty inherent in using a single GCM [92], but the EcoCrop and Maxent models do not cater for the standard deviation between the GCMs. A further assumption is in the use of the representative concentration pathway 8.5, which is a high emission scenario. Using a less aggressive scenario (for example, RCP 2.6, RCP 4.5) would likely reduce the impacts of climate change on climatic crop suitability under the future conditions and this is likely to affect the final crop vulnerability results. Further research could quantify the impacts of using different RCPs on the climate suitability component of the CRVA. Another potential avenue for selecting a future climate scenario is to identify the RCP which is used by the respective ministry in the country of interest, this will likely result in greater uptake of the final vulnerability recommendations.

We also acknowledge that the GIS analysis may possess some biases which influence the final outputs. Notably we have undertaken the study at a policy unit which is fine resolution. It is notable that several of the administrative units which possess smaller areas tend to have higher vulnerability, for example, in the case of Uganda, we see that the sub county of Pader TC, located in the district of Pader, northern Uganda is in the “highest” vulnerability category, whilst all the surrounding sub counties are “high” vulnerability. The sub county is 64 km^2^, which is below the average sub county area (250km^2^), and smaller than the surrounding sub county’s. Nevertheless, besides the adaptive capacity data for poverty and education (district level) the utilised datasets were either at the sub county level or at a resolution of roughly 1km (Table 2, see methods), which therefore enables sub county analysis, including those at <100km^2^.

We have also made assumptions regarding the indicators used to represent the components of vulnerability. A recent study by Cinner et al [109] propose an alternative approach to understanding adaptive capacity that includes the willingness and capability to turn resources into adaptation action and suggest additional criteria, beyond wealth, education and organizational capacity (Table 2) that can be used to determine the capacity to respond to change. We have assumed simplistically that adaptive capacity is homogenous across the analysed cropping systems for the respective countries based on a number of indicators (Table 2). We acknowledge the complexity of capturing adaptive capacity and understand that it may vary depending on the community or farming system being analysed, for instance, it may be that wealthier farmers grow cash crops and therefore have greater resistance to climate change, or that the adaptive capacity of coffee farmers is driven by different indicators to that of rice farmers. Furthermore, we understand that farmers are constantly responding to changes in the climate, whilst in our study we have compiled spatially explicit secondary data on the current adaptive capacity of the population which does not capture the fluid and responsive nature of adaptation to climate change. Our methodology is intended to capture the general patterns of vulnerability to climate change at a national extent through an agricultural lens. We spatially identify the vulnerability of administrative areas to climate change using secondary data, but we do not have the local scale data required to capture the responsiveness of individual farmers to climate stresses. We can accept this as a limitation of our study but one that is necessary due to the pan tropical extent of our research. Future studies could undertake primary data collection at a fine temporal (daily) and spatial resolution (house hold) for administrative areas that we have identified as vulnerable in order to identify how farmers respond on a daily basis to climate challenges. The updates in the conceptualization and measure of adaptive capacity provides an opportunity to develop a more responsive and complex representation of vulnerability that goes beyond the traditional indicators.

## Conclusion

A need exists to identify which areas at the sub national scale are most vulnerable to climate change and require intervention and support. The limited financial and technical capacity available to countries means that investments must be strategic and targeted to the most vulnerable areas. In light of this we have applied the CRVA methodology to Vietnam, Nicaragua and Uganda, three developing countries located in the tropics. In Vietnam we have identified three zones which are particularly vulnerable to climate change, notably (i) the Mekong Delta, due to a loss of climatic suitability for rice and maize (sensitivity) and the presence of flooding, drought and sea level rise (exposure), (ii) the central highlands, due to a loss of climatic suitability for coffee (sensitivity), the presence of flooding and drought (exposure) and low adaptive capacity, and (iii) Son La, due to a loss of climatic suitability for maize (sensitivity), the presence of soil erosion (exposure), and low adaptive capacity.

The proposed CRVA methodology provides a robust and policy relevant insight into the potential impacts of climate change on agricultural systems. It can be used to stimulate debate at the national level in order to determine which areas of the country are most vulnerable to climate change and require strategic investments. Quantifying vulnerability however at a sub-national scale is complex and it is important that the limitations and assumptions are clearly understood by stakeholders and interested policy makers.

Using the latest available data and crop models we have captured the sensitivity of the key crops to climate change, integrated exposure from natural hazards which affect agricultural systems and rural areas, and adopted a broad number of indicators to represent the adaptive capacity of the population, in order to provide a clearer understanding of agricultural vulnerability to climate change. We have built upon previous studies [23] by selecting multiple crops that are key to food and economic security of rural populations and we have developed the concept of vulnerability from previous studies [7] through the integration of natural hazards into the exposure component of vulnerability. The proposed CRVA offer a means to more objectively prioritise climate change adaptation policies which are strategically targeted to the most vulnerable rural communities. This is particularly important due to the limited technical and financial resources available to confront the mammoth impacts of climate change on rural communities in developing tropical countries.

## Supporting information

S1 Table Summary of studies that use GIS analysis to quantify vulnerability of rural communities to climate change. The table offers insights into the key characteristics of the studies In order to place the conceptual framework of vulnerability in the reported studies against the CRVA approach outlined in this paper.
(DOCX)

S2 Table Methodology used to quantify potential soil erosion impact in Vietnam. The Revised Universal Soil Loss Equation (RUSLE) [112] was applied using local datasets for the respective erosion factors.
(DOCX)

S3 Table Indicators used to capture Adaptive Capacity in Nicaragua. Selected indicators and assigned weights are adopted from the Bouroncle et al [7] study.
(DOCX)

S4 Table A summary of selected vulnerable regions for Nicaragua (NIC) and Vietnam (VNM), with a reference to the “main drivers” (Sensitivity and Exposure) behind the vulnerability and the respective Adaptive Capacity (AC). A crop is classified as sensitive to climate change when climate suitability decline is equivalent to an index (see Table 3) of 0.25 (-5 - -24%), 0.5 (-25 - -49%) or 1 (-50 - -100%) for the respective administrative area. An indicator is listed in Exposure when the administrative area is in the top 25% of most affected administrative areas for the respective indicator (Nicaragua: Highest (1)–Lowest (154), Vietnam: Highest (1)–Lowest (692)) with the rank documented in the table. Adaptive Capacity (AC) is shown as a single index for Nicaragua documented as Low (0–0.3), medium (0.3–0.6) and high (0.6–1). In Vietnam, AC Index is displayed as low (0.535–0.661), medium (0.662–0.771) and high (0.772–1) and also the individual indicators which are in the bottom 25% when ranked lowest to highest with the recorded rank are documented (Vietnam Lowest (1)–Highest (692)).
(DOCX)

S1 Fig Adaptive capacity map for the case study country: Vietnam. The respective indicators (Poverty, Health, Infrastructure….) are displayed in 3 classes (low, medium, high) based on the natural breaks (jenks) classification using ArcMap 10.1. Overall AC Index is displayed as low (0.535–0.661), medium (0.662–0.771) and high (0.772–1) corresponding to the bottom, middle and highest third when administrative areas are ranked from lowest to highest AC.
(TIF)

S2 Fig Vulnerability of Rice and Coffee (Robusta) to climate change (2050) under a high emission scenario (RCP 8.5). The vulnerability of rice and coffee (Robusta) are a function of sensitivity, exposure and adaptive capacity. The vulnerability index is categorized into five classes from ‘lowest’ to ‘highest’ using the equal intervals classification in ArcMap 10.1.
(TIF)

S1 File Crop parameters, model and selected natural hazards for Nicaragua, Uganda and Vietnam. The MaxEnt Model [110] was used for selected crops when accurate presence data was available, or if the crop is predominantly irrigated, such is the case of Rice in Vietnam [111] or grown at particular elevational ranges, for example, coffee Arabica in Nicaragua and coffee Robusta in Vietnam. The Ecocrop model was used when geographic distribution data (presence data) was not available or reliable. The natural hazards were selected and verified by experts in Vietnam and Nicaragua.
(XLSX)
