# Supplementary material for: Vulnerability of the agricultural sector to climate change: The development of a pan-tropical Climate Risk Vulnerability Assessment to inform sub-national decision making
Source: PLoS One. 2019 Mar 27;14(3):e0213641. doi: 10.1371/journal.pone.0213641 (PMC6436735; doi:10.1371/journal.pone.0213641)
Supplement: S1 Table — The table offers insights into the key characteristics of the studies In order to place the conceptual framework of vulnerability in the reported studies against the CRVA approach outlined in this paper. (DOCX) [file pone.0213641.s001.docx]

| Reference/ year | Purpose | Number of crops analysed | Spatial Extent | Vulnerability focus and analysis | Spatial Resolution | Temporal Scale |
| --- | --- | --- | --- | --- | --- | --- |
| Baca et al (2014) | Assess vulnerability of coffee farmers to climate change | coffee | Mesoamerica | IPCC Vulnerability  Vulnerability of coffee farm productions zones to climate change  Exposure: Change in Crop suitability under climate change.  Sensitivity and Adaptive Capacity combined: Included- Food and health, Migration, Yield Variability, Quality of housing …. | 1 km | Current, 2050 |
| Sehgal et al (2013) | Assess the vulnerability of agriculture to climate change | Not crop specific | Indo-Gangetic plain | IPCC Vulnerability  Vulnerability of agricultural areas to climate change  Exposure:  Change in Temperature and precipitation (1951-2009)  Sensitivity:  Presence of sown area, productivity of food grains, organic carbon content of soil  Adaptive Capacity:  % Irrigated land area, Human Development Index, Cropping intensity,  Livestock density | District^1^ | Current (temperature and precipitation 1951 – 2009) |
| Torresan et al (2012) | Assessment of coastal vulnerability to climate change hazards | 0 | North Adriatic Sea | Natural – human system  Number of indicators incorporating socio-economic capacity and biophysical risk | 25m | Current |
| Berry et al (2006) | Assessment of vulnerability of agricultural area and species richness to climate change | 0 | Western Europe | Vulnerability indicators based on Accelerates LandUse model | District | Current, 2050 |
| Yusuf and Francisco (2009) | Climate change vulnerability mapping in SE Asia | 0 | SE Asia | IPPC Vulnerability  Exposure:  Presence of biophysical risks (Tropical Cyclone, Drought, Flood, Landslide, Sea Level Rise)  Sensitivity:  Population density & Protected Areas  Adaptive Capacity :  Socio economic capacity, technology, infrastructure | Province^2^ | Current |
| Lenin Corrales et al (2012) | Climate change analysis of natural and social systems in coastal areas of Central America | 0 (not focused on agriculture) | Belize, Guatemala, Honduras (Caribbean coast) | IPCC Vulnerability  Vulnerability of coastal communities to:   - Change in temperature and precipitation - Change in sea suface temperature - Sea level rise | District | Current, Future (2030,2050,2070,2100) |
| Bouroncle et al (2017) | Mapping vulnerability of small holder agricultural livelihoods in Central America | coffee, maize, beans, upland rice, sorghum, plantain, and cassava | Guatemala, El Salvador, Honduras and Nicaragua | IPCC definition  Assesses the impacts of changes in climatic suitability of the analysed crops as a function of the communities (district) adaptive capacity | District | Current, 2030 |
