# Supplementary material for: Vulnerability of the agricultural sector to climate change: The development of a pan-tropical Climate Risk Vulnerability Assessment to inform sub-national decision making
Source: PLoS One. 2019 Mar 27;14(3):e0213641. doi: 10.1371/journal.pone.0213641 (PMC6436735; doi:10.1371/journal.pone.0213641)
Supplement: S2 Table — The Revised Universal Soil Loss Equation (RUSLE) [112] was applied using local datasets for the respective erosion factors. (DOCX) [file pone.0213641.s002.docx]

| Factors | Source |
| --- | --- |
| A: annual soil loss rate (ton /ha/yr) | - |
| R: rainfall factor (MJ.mm/ha.yr) | Fick et al [1]; Bien Le Van [2] |
| K: soil erodibility factor (ton.ha.h / MJ.ha.mm) | Ashiagbor [3]; Ranzi et al [3]; da Silva et al [4]; GAEZ v3.0 [4] |
| LS: is slope steepness and slope length factor (dimensionless) | DEM - SRTM [4] |
| C: cover factor (dimensionless) | C factor from Morgan [5] Land cover from USGS [6] |
| P: conservation practices (dimensionless) | * No information, P = 1 |

**References**

1. Fick SE, Hijmans RJ. Worldclim 2: New 1-km spatial resolution climate surfaces for global land areas. International Journal of Climatology [Internet]. 2017 [cited 12 Nov 2016]. Available: http://worldclim.org/version2

2. Le Van B, Truong Phuoc M, Tran Thi A, Raghavan V. An open source GIS approach for soil erosion modeling in Danang City, Vietnam. 2014;

3. Ashiagbor G, Forkuo EK, Laari P, Aabeyir R. Modeling soil erosion using RUSLE and GIS tools. International Journal of Remote Sensing & Geoscience. 2013;2: 1–17.

4. Jarvis A, Reuter IH, Nelson A, Guevera E. Hole-filled SRTM for the globe Version 4, available from the CGIAR-CSI SRTM 90m Database [Internet]. 2008. Available: http://www.cgiar-csi.org/data/srtm-90m-digital-elevation-database-v4-1

5. Morgan R. Soil, erosion and conservation. 3rd ed. Australia: Blackwell Publishing; 2005.

6. Broxton PD, Zeng X, Sulla-Menashe D, Troch PA. A Global Land Cover Climatology Using MODIS Data. Journal of Applied Meteorology and Climatology. 2014;53: 1593–1605. doi:10.1175/JAMC-D-13-0270.1
