# Supplementary material for: Vulnerability of the agricultural sector to climate change: The development of a pan-tropical Climate Risk Vulnerability Assessment to inform sub-national decision making
Source: PLoS One. 2019 Mar 27;14(3):e0213641. doi: 10.1371/journal.pone.0213641 (PMC6436735; doi:10.1371/journal.pone.0213641)
Supplement: S3 Table — Selected indicators and assigned weights are adopted from the Bouroncle et al [7] study. (DOCX) [file pone.0213641.s003.docx]

| Adaptive capacity condition | Criteria (weight) | **Indicator** |
| --- | --- | --- |
| Satisfaction of basic needs | Safe drinking water (0.33) | Rural households with access to safe drinking water |
|  | Public health (0.27) | Primary healthcare units per 1000 people |
|  | Education (0.20) | Rural school-aged population (aged 7-17) that attends school |
|  | Housing (0.13) | Rural dwellings built with long-lasting materials |
|  | Equity (0.07) | Rural Gender Parity Index |
| Resources for innovation | Land (0.50) | Entitled agricultural production units |
|  | Technical assistance (0.33) | Agricultural production units that received technical assistance |
|  | Infrastructure (0.17) | Roads density |
| Resources for action | Financial resources (0.66) | Rural economically active population employed in non-agricultural activities |
|  |  | Agricultural production units that received a loan |
|  | Labour Force (0.34) | Rural Demographic dependency ratio |
