# Supplementary material for: Vulnerability of the agricultural sector to climate change: The development of a pan-tropical Climate Risk Vulnerability Assessment to inform sub-national decision making
Source: PLoS One. 2019 Mar 27;14(3):e0213641. doi: 10.1371/journal.pone.0213641 (PMC6436735; doi:10.1371/journal.pone.0213641)
Supplement: S4 Table — A crop is classified as sensitive to climate change when climate suitability decline is equivalent to an index (see Table 3) of 0.25 (-5 - -24%), 0.5 (-25 - -49%) or 1 (-50 - -100%) for the respective administrative area. An indicator is listed in Exposure when the administrative area is in the top 25% of most affected administrative areas for the respective indicator (Nicaragua: Highest (1)–Lowest (154), Vietnam: Highest (1)–Lowest (692)) with the rank documented in the table. Adaptive Capacity (AC) is shown as a single index for Nicaragua documented as Low (0–0.3), medium (0.3–0.6) and high (0.6–1). In Vietnam, AC Index is displayed as low (0.535–0.661), medium (0.662–0.771) and high (0.772–1) and also the individual indicators which are in the bottom 25% when ranked lowest to highest with the recorded rank are documented (Vietnam Lowest (1)–Highest (692)). (DOCX) [file pone.0213641.s004.docx]

| **Country** | **Region** | **Administrative area(s)** | **Main Drivers** | | |
| --- | --- | --- | --- | --- | --- |
|  |  |  | **Sensitivity** | **Exposure** | **AC** |
| Nicaragua | 1 | Siuna | Rice (0.25) Coffee (0.25) Cacao (0.25) Bean (0.5) | Tropical Cyclones (17) | Low AC |
|  |  | Bonanza | Rice (0.5), Maize (0.25), Cacao (0.5), Beanapan (0.5) | Tropical Cyclones (13) | Low AC |
|  | 2 | Rancho Grande | Rice (0.25), Coffee (0.5), Cacao (0.25), Beanpost (0.25), Beanapan (0.5) | soil erosion (35) | Low AC |
|  |  | Matiguas | Rice (0.25), Coffee (0.5), Cacao (0.25), Beanpost (0.5), Beanapost (0.5) | Flooding (30) | Low AC |
|  |  | Muy Muy | Rice (0.5), Maize (0.25), Coffee (0.5), Cocoa (0.25), Beanpost (0.5), Beanapan (0.5) | Flooding (12) | Low AC |
|  |  | Boaco | Rice (0.25), Coffee (0.5), ), Cocoa (0.25), Beanpost (0.5), Beanapan (0.5) | Flooding (38) | Low AC |
|  | 3 | Acoyapa | Rice (0.5), Cocoa (0.5), Beanpost (0.5), Beanapan (0.5) | Tropical Cyclone (25) | Low AC |
|  |  | Villa Sandino | Rice (0.25), Maize (0.25), Cacao (0.5), Beanapan (0.5) | Tropical Cyclone (18) | Low AC |
|  |  | El Almendro | Rice (0.25), Maize (0.25), Cacao (0.5), Beanapan (1) | Tropical Cyclone (19) | Low AC |
|  |  | El Castillo | Rice (0.5), Maize (0.25), Cacao (1), Beanapan (0.5) | Tropical Cyclone (12)  Flooding (41) | Low AC |
| Vietnam | Son La | Bac Yen |  | Soil erosion (1) | Medium AC  Poverty (56)  Health (146)  Education (17) |
|  |  | Muong La |  | Soil erosion (2) | Medium AC  Poverty (69)  Health (147)  Infrastructure (126)  Education (18) |
|  |  | Quỳnh Nhai |  | Soil erosion (3) | Medium AC  Health (151)  Infrastructure (104)  Education (22) |
|  | Central Highlands | Chu Pah (Gia Lai Province) | Coffee (0.25) | Flooding (31)  Drought (67) | Low AC  Poverty (53)  Health (63)  Education (59) |
|  |  | Ea Súp (Dak Lak Province) | Coffee (0.25) | Drought (83) | Low AC  Poverty (119),  Health (41),  Education (4) |
|  |  | Kon Rẫy (Kon Tum Province) | Coffee (0.25) | Erosion (68) | Low AC  Poverty (12),  Health (5),  Infrastructure (161) |
|  |  | Cu Jút (Dac Nong Province) | Coffee (0.5) | Erosion (103) | Low AC  Poverty (144),  Health (52),  Infrastructure (91),  Organisational Capacity (97),  Education (75) |
|  |  | Ðam Rông (Lam Dong Province) | Coffee (0.25) | Erosion (139) | Low AC  Poverty (17), |
|  | Mekong River Delta | Phụng Hiệp (Hau Giang Province) | Rice (0.25), Maize (0.5) | Sea level rise (39)  Drought (89)  Flooding (84) | High AC |
|  |  | My Tu (Sóc Trang province) | Maize (0.25) | Sea Level Rise (12)  Drought (44)  Flooding (96) | Medium AC |
|  |  | Tran De (Sóc Trăng Province) | Rice (0.25), Maize (0.25) | Sea Level Rise (57)  Drought (124) | Medium AC |
|  |  | Phuoc Long (Bạc Liêu Province) | Rice (0.25) | Sea Level Rise (32)  Drought (144)  Flooding (58) | Medium AC |
