# Supplementary figures and images for: Vulnerability of the agricultural sector to climate change: The development of a pan-tropical Climate Risk Vulnerability Assessment to inform sub-national decision making
Source: PLoS One. 2019 Mar 27;14(3):e0213641. doi: 10.1371/journal.pone.0213641 (PMC6436735; doi:10.1371/journal.pone.0213641)

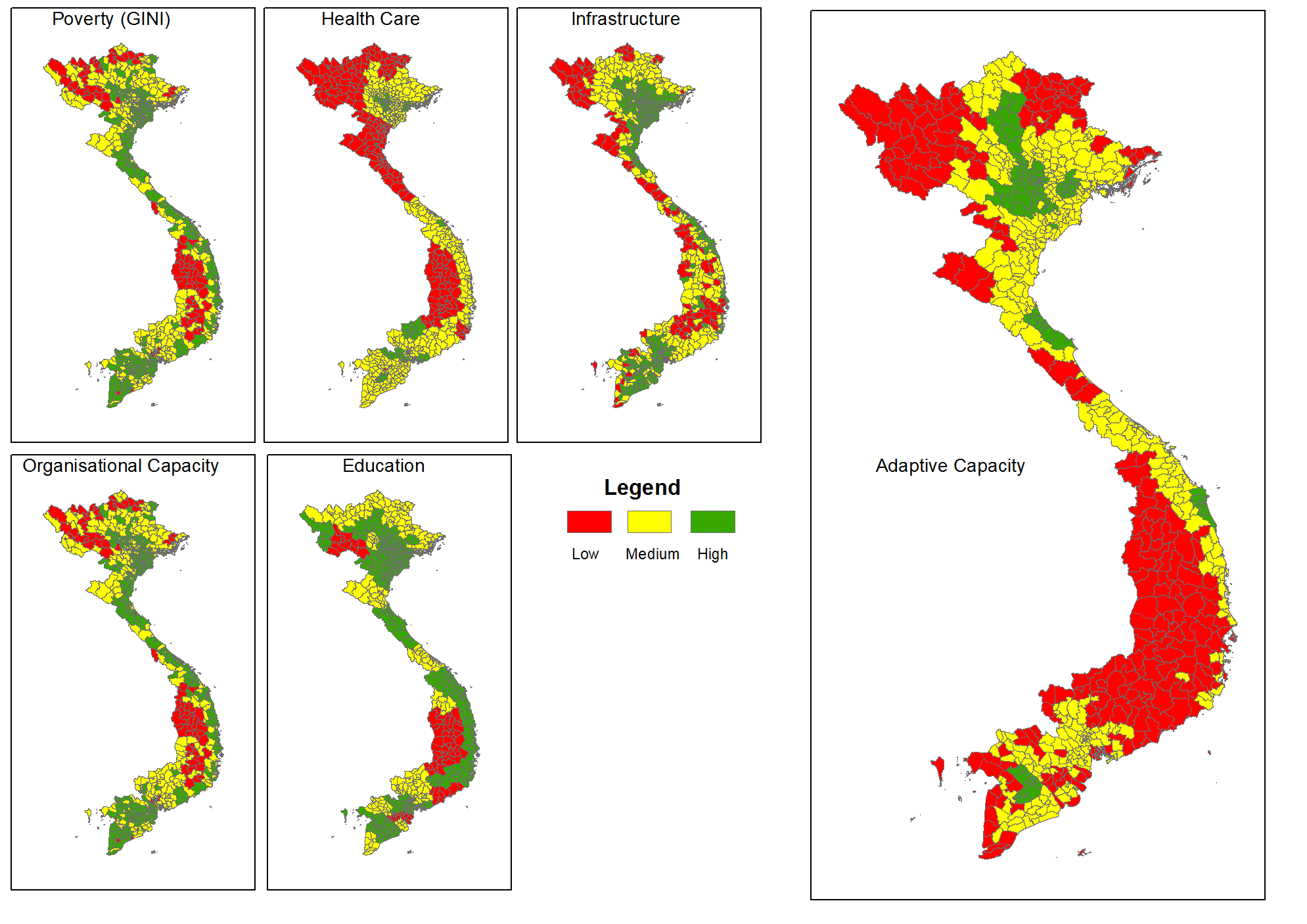

Supplement: S1 Fig — The respective indicators (Poverty, Health, Infrastructure….) are displayed in 3 classes (low, medium, high) based on the natural breaks (jenks) classification using ArcMap 10.1. Overall AC Index is displayed as low (0.535–0.661), medium (0.662–0.771) and high (0.772–1) corresponding to the bottom, middle and highest third when administrative areas are ranked from lowest to highest AC. (TIF) [file pone.0213641.s005.tif]

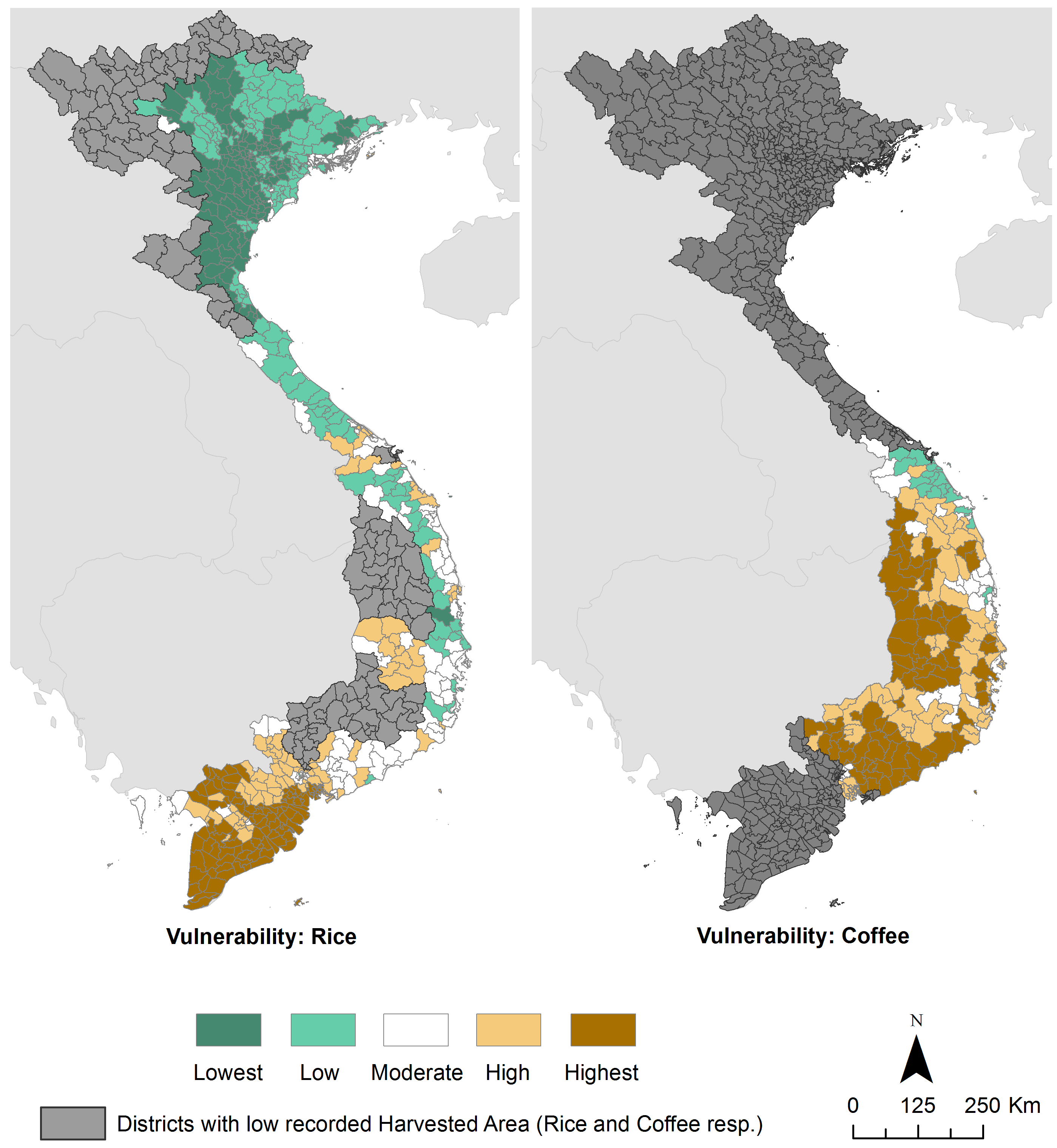

Supplement: S2 Fig — The vulnerability of rice and coffee (Robusta) are a function of sensitivity, exposure and adaptive capacity. The vulnerability index is categorized into five classes from ‘lowest’ to ‘highest’ using the equal intervals classification in ArcMap 10.1. (TIF) [file pone.0213641.s006.tif]
